# Supplementary material for: Visual Clustering of Transcriptomic Data from Primary and Metastatic Tumors—Dependencies and Novel Pitfalls
Source: Genes (Basel). 2022 Jul 26;13(8):1335. doi: 10.3390/genes13081335 (PMC9394300; doi:10.3390/genes13081335)
Supplement: Supplementary file 1 [file genes-13-01335-s001.zip › genes-1693305-supplementary.pdf]

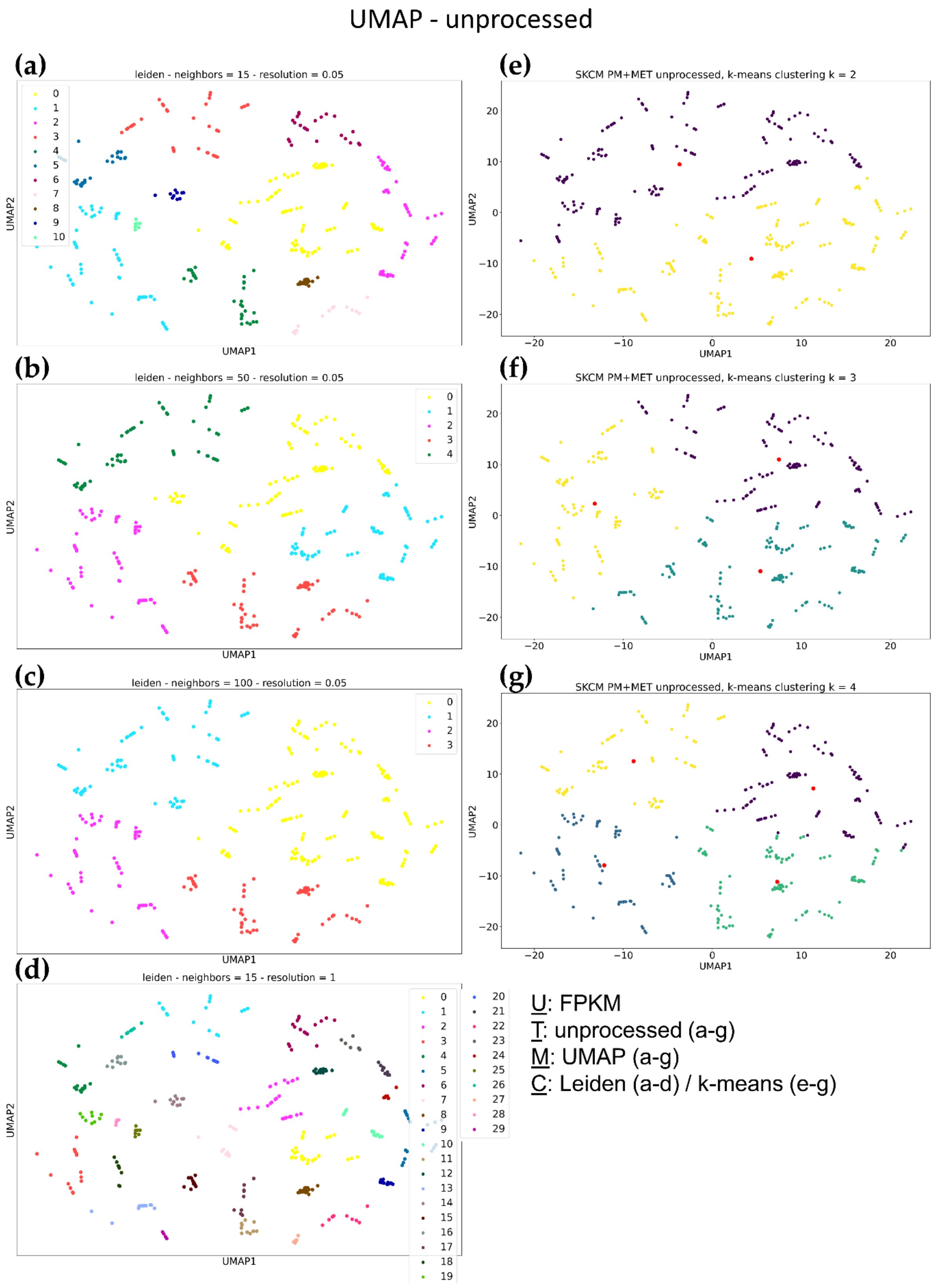

Figure S1: Different clustering approaches for unprocessed TCGA-SKCM data. Clustering of the complete TCGA-SKCM dataset consisting of primary tumors and metastases using UMAP with untransformed data, analogous to Figure 5d with different clustering algorithms and parameters. Leiden clustering with (a) n_neighbors = 15 and resolution = 0.05, (b)

n_neighbors = 50 and resolution = 0.05, (c) n_neighbors = 100 and resolution = 0.05, and (d) n_neighbors = 15 and resolution = 1. k-means clustering with (e) k = 2, (f) k = 3, and (g) k = 4. The red dot marks the centre of the respective cluster.

FPKM: Fragments Per Kilobase Million; U: Unit, T: Transformation, M: data dimension reduction Method, C: Clustering method.

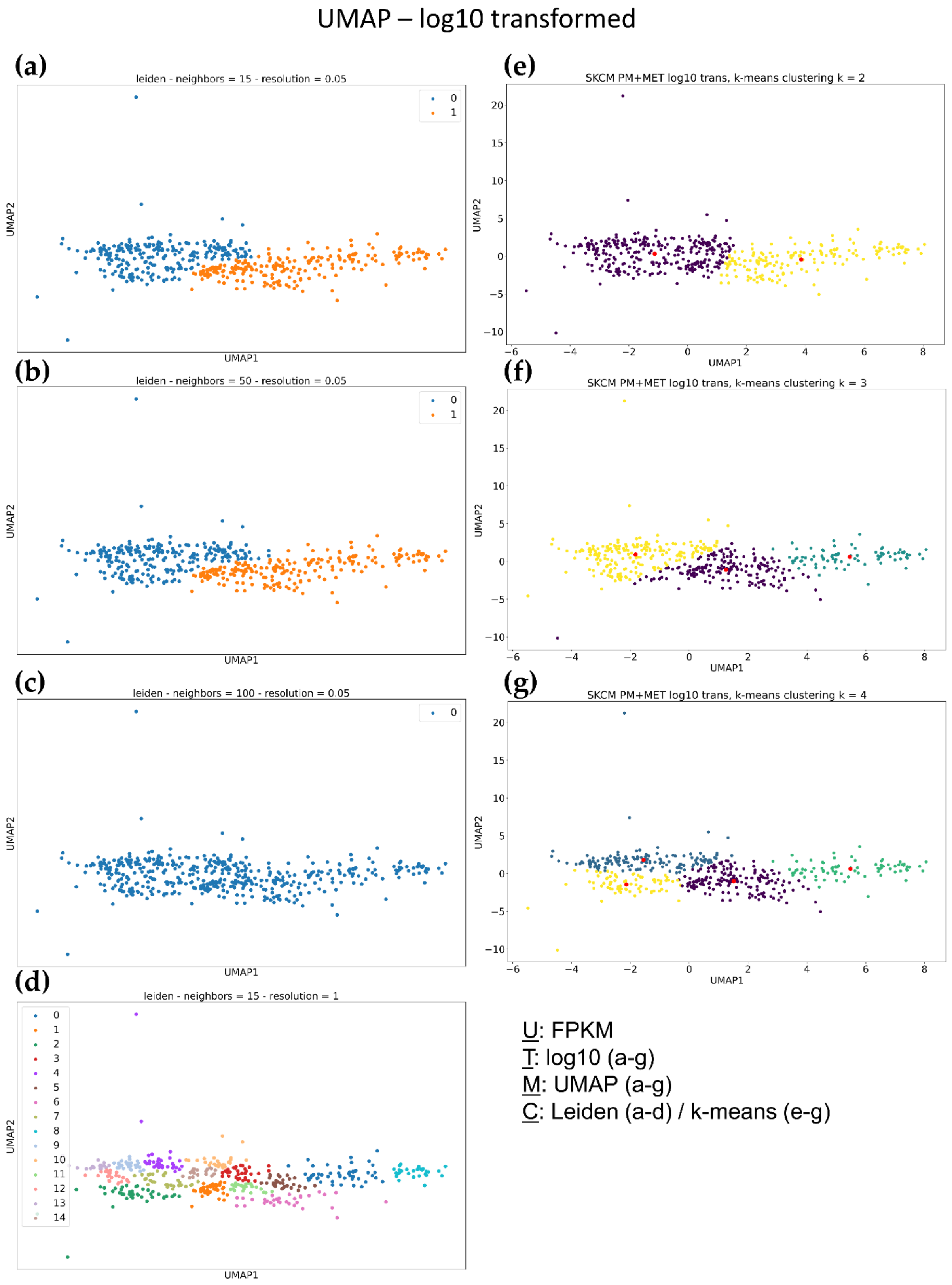

Figure S2: Different clustering approaches for log10 transformed TCGA-SKCM data. Clustering of the complete TCGA-SKCM dataset consisting of primary tumors and metastases using UMAP with log10 transformed data, analogous to Figure 5e with different clustering algorithms and parameters. Leiden clustering with (a) n_neighbors = 15 and resolution = 0.05, (b) n_neighbors = 50 and resolution = 0.05, (c) n_neighbors = 100 and resolution = 0.05, and (d)

n_neighbors = 15 and resolution = 1. k-means clustering with (e) k = 2, (f) k = 3, and (g) k = 4. The red dot marks the centre of the respective cluster.

FPKM: Fragments Per Kilobase Million; U: Unit, T: Transformation, M: data dimension reduction Method, C: Clustering method.

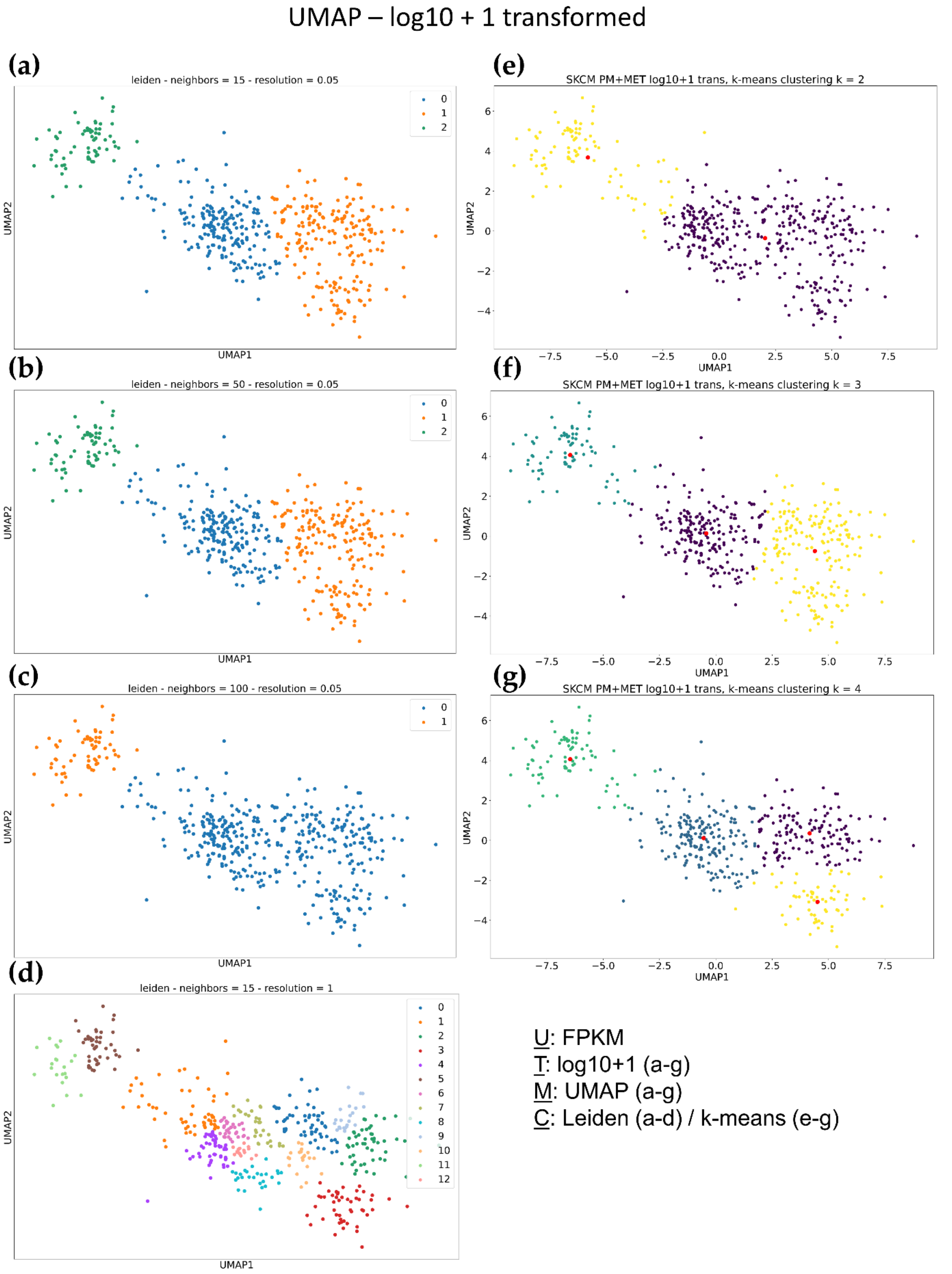

Figure S3: **Different clustering approaches for log10 + 1 transformed TCGA-SKCM data.** Clustering of the complete TCGA-SKCM dataset consisting of primary tumors and metastases using UMAP with log10 + 1 transformed data, analogous to Figure 5f with different clustering algorithms and parameters. Leiden clustering with (a) n_neighbors = 15 and resolution = 0.05,

(b) n_neighbors = 50 and resolution = 0.05, (c) n_neighbors = 100 and resolution = 0.05, and (d) n_neighbors = 15 and resolution = 1. k-means clustering with (e) k = 2, (f) k = 3, and (g) k = 4. The red dot marks the centre of the respective cluster.

FPKM: Fragments Per Kilobase Million; U: Unit, T: Transformation, M: data dimension reduction Method, C: Clustering method.

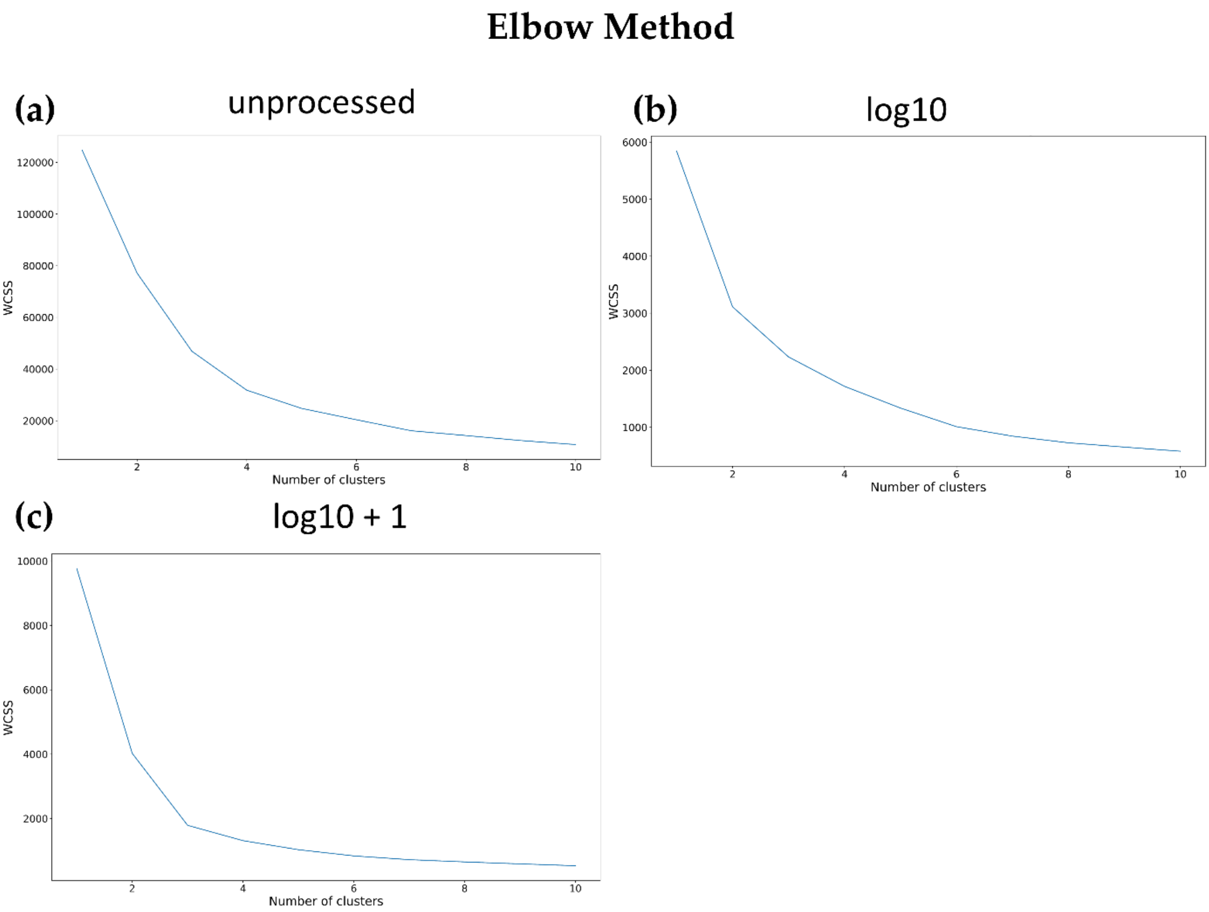

Figure S4: **Elbow Method for k-means clustering of the TCGA-SKCM dataset.** Results of the performed elbow method using the KMeans function of the sklearn.cluster method, displaying the inertia for different amounts of clusters (1-10) for the UMAP results of the whole TCGA-SKCM dataset – based on (a) unprocessed, (b) log10 transformed, and (c) log10 + 1 transformed data.

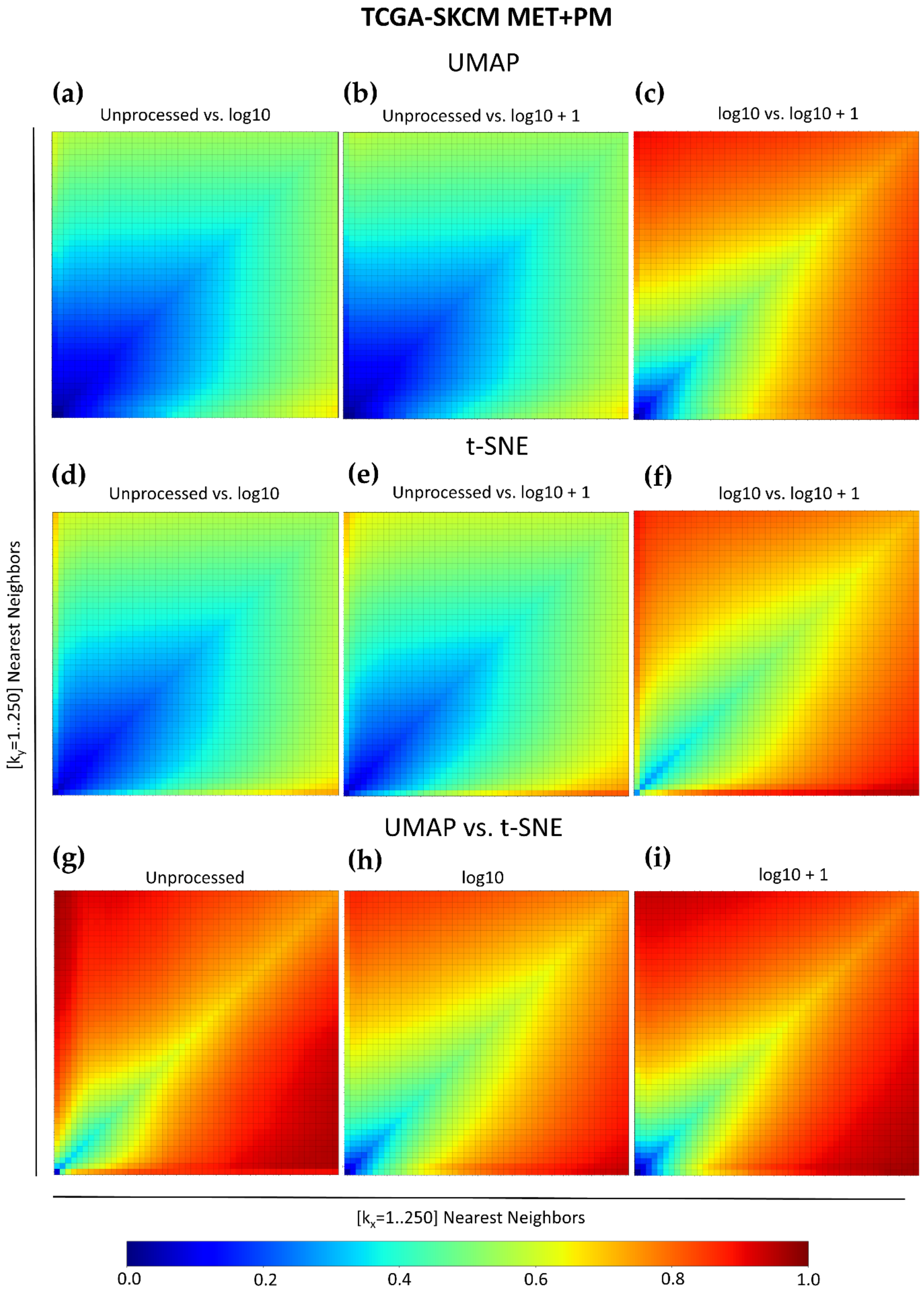

TCGA-SKCM MET+PM
UMAP
(a)
Unprocessed vs. log10
(b)
Unprocessed vs. log10 + 1
(c)
log10 vs. log10 + 1
t-SNE
(d)
Unprocessed vs. log10
(e)
Unprocessed vs. log10 + 1
(f)
log10 vs. log10 + 1
UMAP vs. t-SNE
(g)
Unprocessed
(h)
log10
(i)
log10 + 1
[k_y=1..250] Nearest Neighbors
[k_x=1..250] Nearest Neighbors
0.0
0.2
0.4
0.6
0.8
1.0

Figure S5: **Flameplots of complete TCGA-SKCM dataset.** Quantification comparison of local similarities for k_{xy}=[1..250] nearest neighbors across two different maps derived from t-SNE or UMAP representation of the complete TCGA-SKCM dataset containing metastasis and primary tumors. Comparison of UMAPs for (a) unprocessed and log10 transformed, (b) unprocessed and log10 + 1 transformed and (c) log10 and log10 + 1 transformed data. Comparison of t-SNE plots for (d) unprocessed and log10 transformed, (e) unprocessed and log10 + 1 transformed and (f) log10 and log10 + 1 transformed data. Comparison of UMAPs and t-SNE plots for (g) unprocessed, (h) log10, and (i) log10 + 1 transformed data.

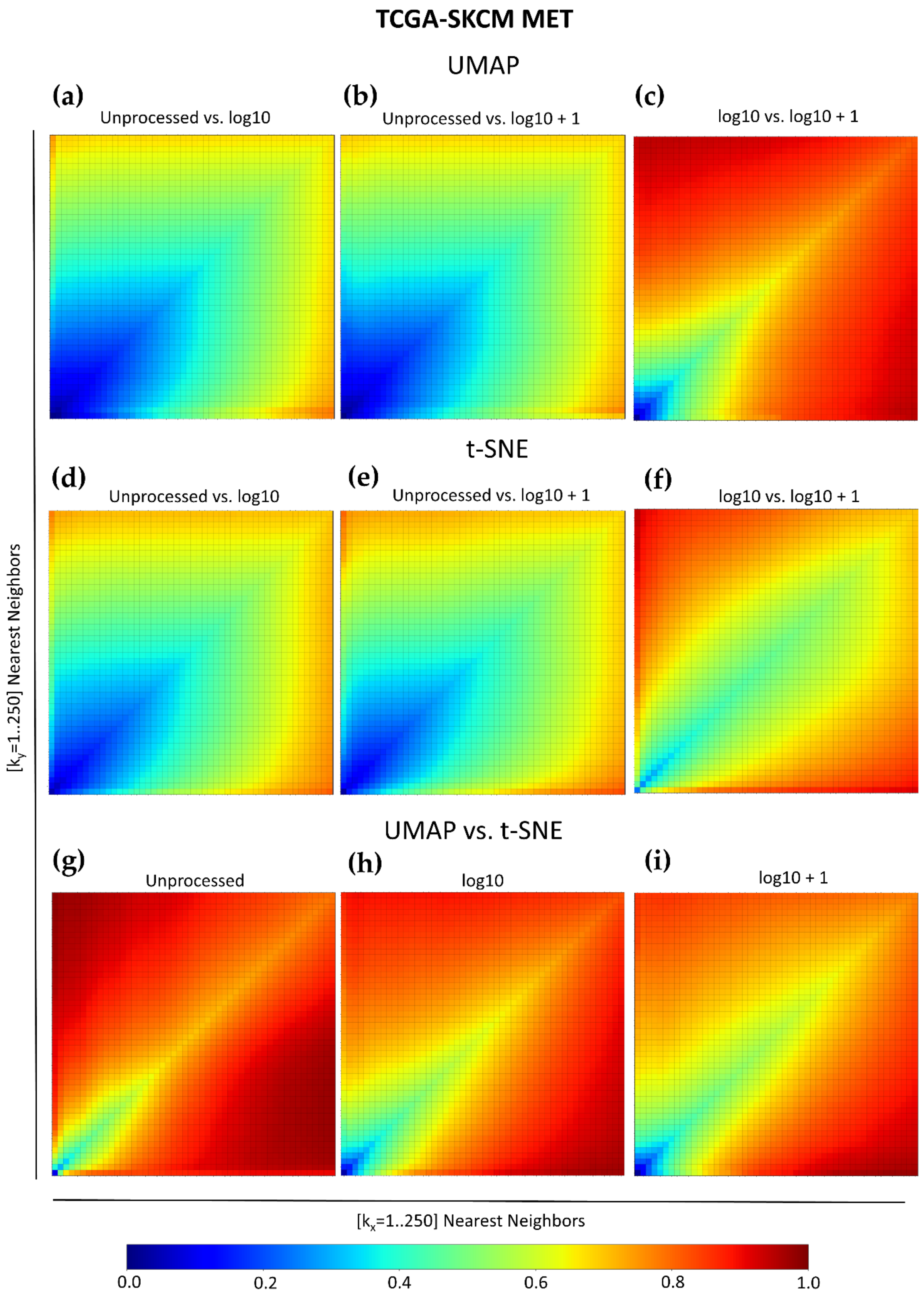

Figure S6: **Flameplots of metastatic samples of TCGA-SKCM dataset.** Quantification comparison of local similarities for k_{xy}=[1..250] nearest neighbors across two different maps derived from t-SNE or UMAP representation of the TCGA-SKCM dataset containing only metastasis. Comparison of UMAPs for (a) unprocessed and log10 transformed, (b)

unprocessed and log10 + 1 transformed and (c) log10 and log10 + 1 transformed data. Comparison of t-SNE plots for (d) unprocessed and log10 transformed, (e) unprocessed and log10 + 1 transformed and (f) log10 and log10 + 1 transformed data. Comparison of UMAPs and t-SNE plots for (g) unprocessed, (h) log10, and (i) log10 + 1 transformed data.

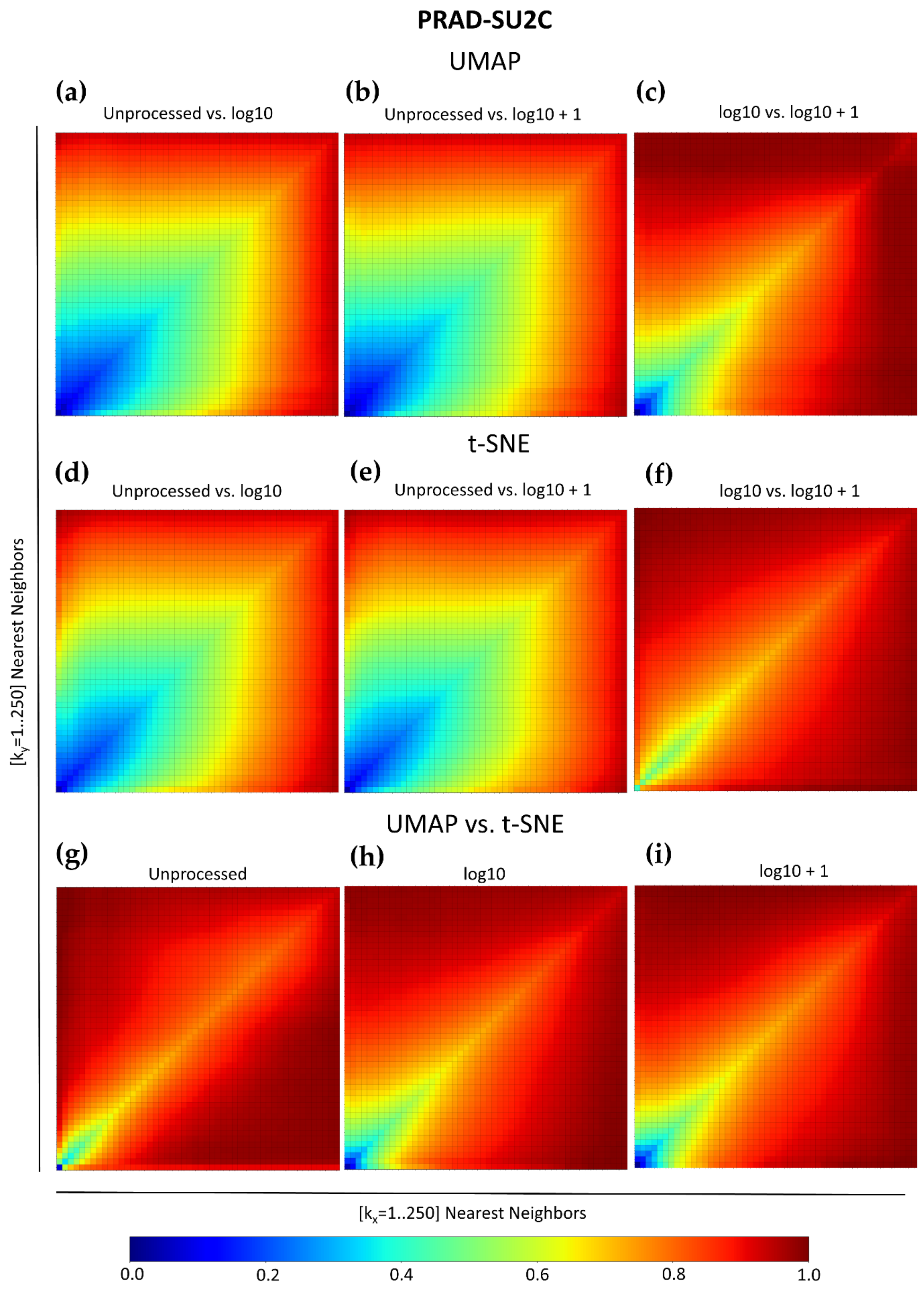

Figure S7: **Flameplots of PRAD-SU2C dataset.** Quantification comparison of local similarities for k_{xy}=[1..250] nearest neighbors across two different maps derived from t-SNE or UMAP representation of the PRAD-SU2C (Dreamteam) dataset. Comparison of UMAPs for (a) unprocessed and log10 transformed, (b) unprocessed and log10 + 1 transformed and (c) log10

and log10 + 1 transformed data. Comparison of t-SNE plots for (d) unprocessed and log10 transformed, (e) unprocessed and log10 + 1 transformed and (f) log10 and log10 + 1 transformed data. Comparison of UMAPs and t-SNE plots for (g) unprocessed, (h) log10, and (i) log10 + 1 transformed data.

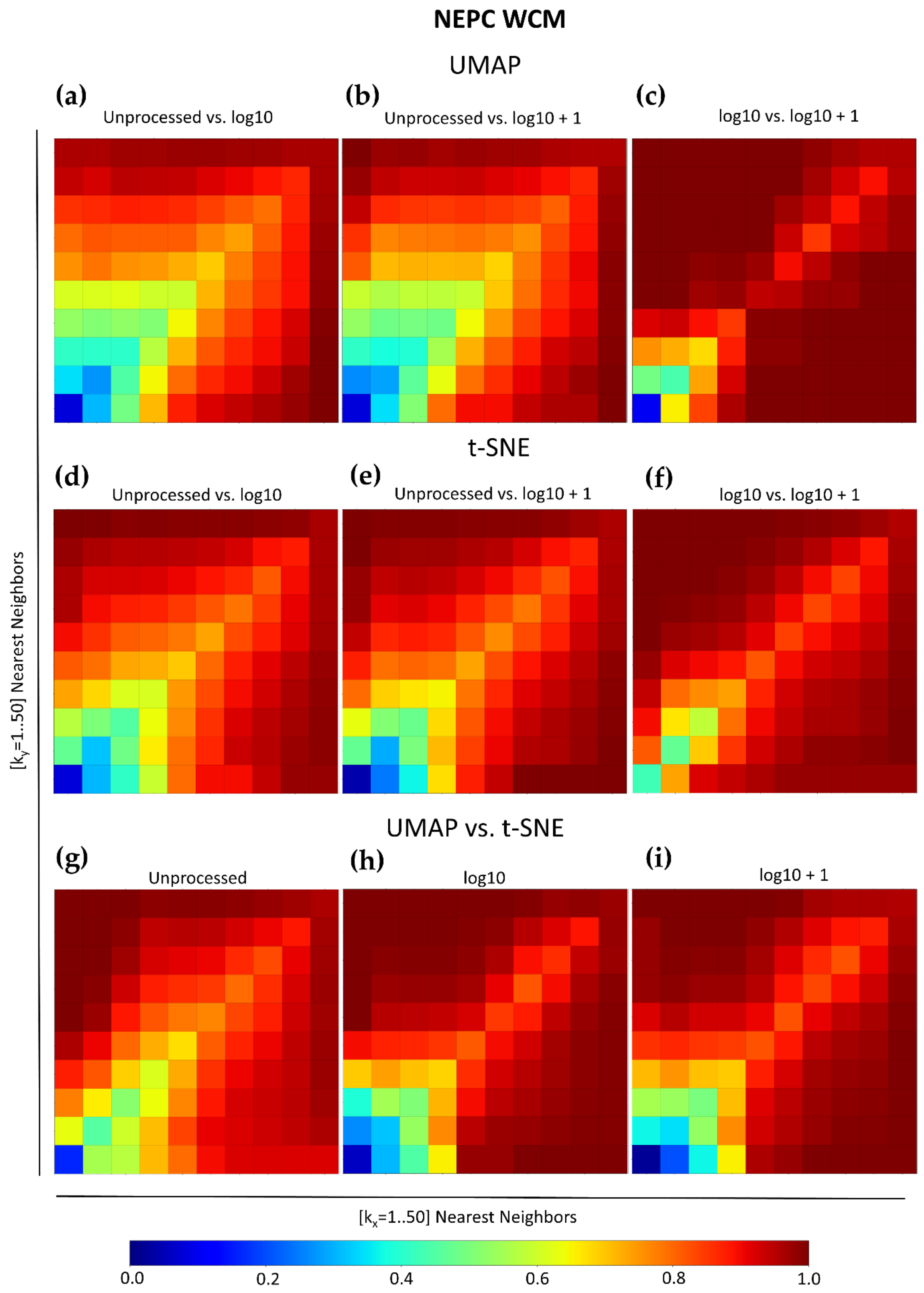

Figure S8: **Flameplots of NEPC WCM dataset.** Quantification comparison of local similarities for k_{xy}=[1..50] nearest neighbors across two different maps derived from t-SNE or UMAP representation of the NEPC WCM dataset. Comparison of UMAPs for (a) unprocessed and log10 transformed, (b) unprocessed and log10 + 1 transformed and (c) log10 and log10 + 1

transformed data. Comparison of t-SNE plots for (d) unprocessed and log10 transformed, (e) unprocessed and log10 + 1 transformed and (f) log10 and log10 + 1 transformed data. Comparison of UMAPs and t-SNE plots for (g) unprocessed, (h) log10, and (i) log10 + 1 transformed data.

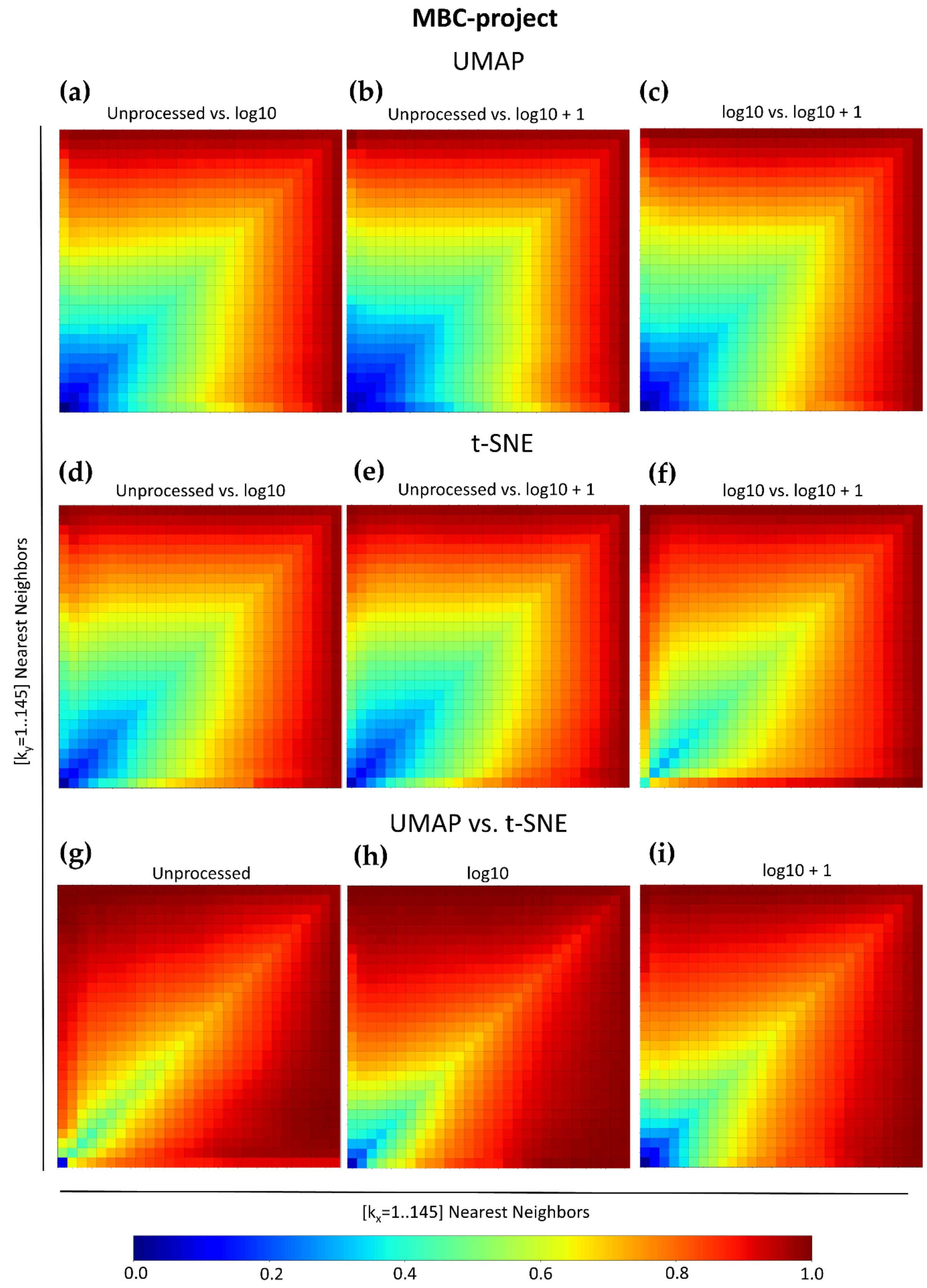

Figure S9: **Flameplots of MBC-project dataset.** Quantification comparison of local similarities for k_{xy}=[1..145] nearest neighbors across two different maps derived from t-SNE or UMAP representation of the MBC-project dataset. Comparison of UMAPs for (a) unprocessed and log10 transformed, (b) unprocessed and log10 + 1 transformed and (c) log10 and log10 + 1

transformed data. Comparison of t-SNE plots for (d) unprocessed and log10 transformed, (e) unprocessed and log10 + 1 transformed and (f) log10 and log10 + 1 transformed data. Comparison of UMAPs and t-SNE plots for (g) unprocessed, (h) log10, and (i) log10 + 1 transformed data.

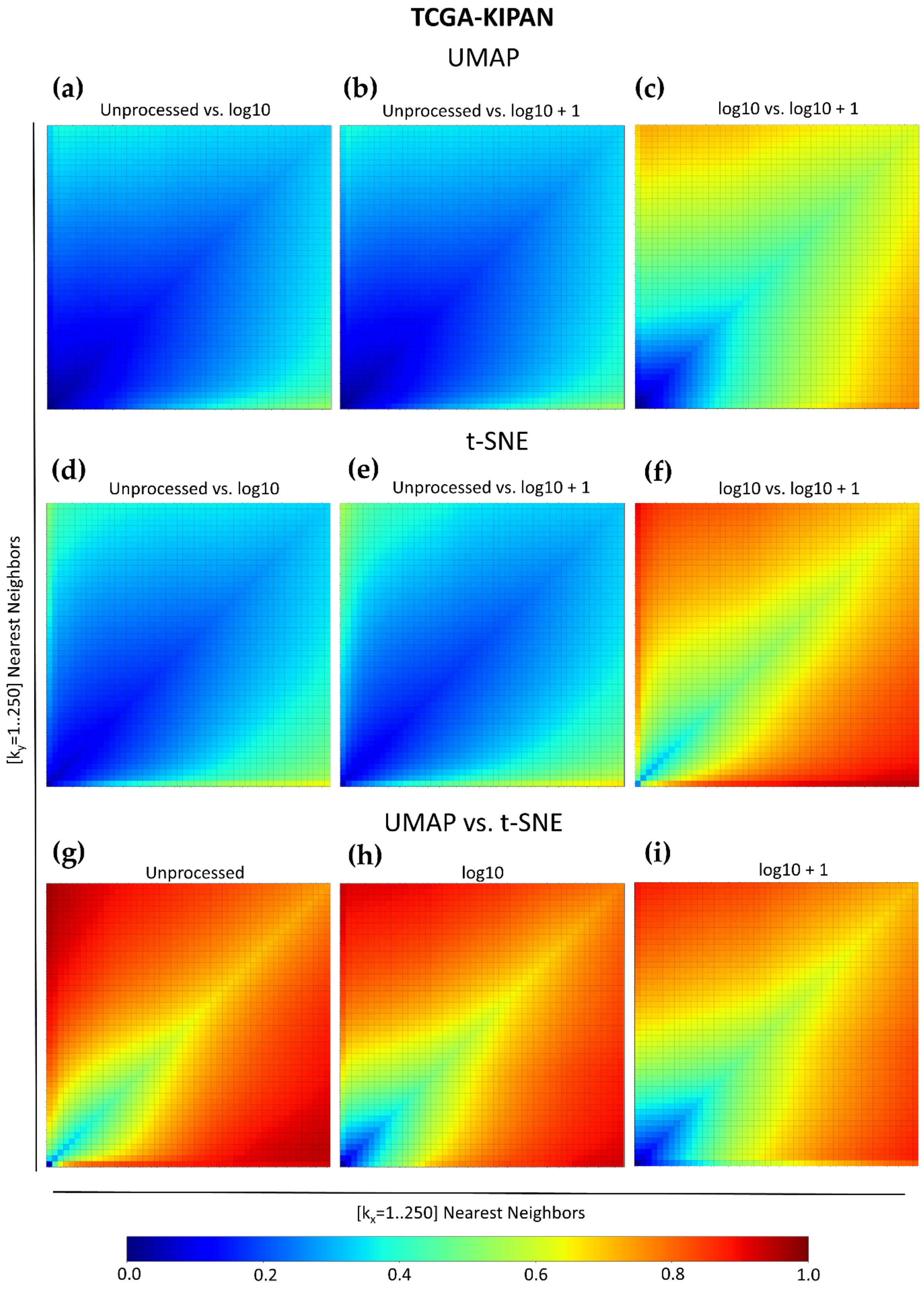

Figure S10: **Flameplots of TCGA-KIPAN dataset.** Quantification comparison of local similarities for k_{xy}=[1..250] nearest neighbors across two different maps derived from t-SNE or UMAP representation of the TCGA-KIPAN dataset, consisting of the three different subgroups clear cell renal cell carcinoma (TCGA-KIRC), papillary renal cell carcinoma (TCGA-

KIRP), and chromophobe renal cell carcinoma (TCGA-KICH). Comparison of UMAPs for (a) unprocessed and log10 transformed, (b) unprocessed and log10 + 1 transformed and (c) log10 and log10 + 1 transformed data. Comparison of t-SNE plots for (d) unprocessed and log10 transformed, (e) unprocessed and log10 + 1 transformed and (f) log10 and log10 + 1 transformed data. Comparison of UMAPs and t-SNE plots for (g) unprocessed, (h) log10, and (i) log10 + 1 transformed data.
